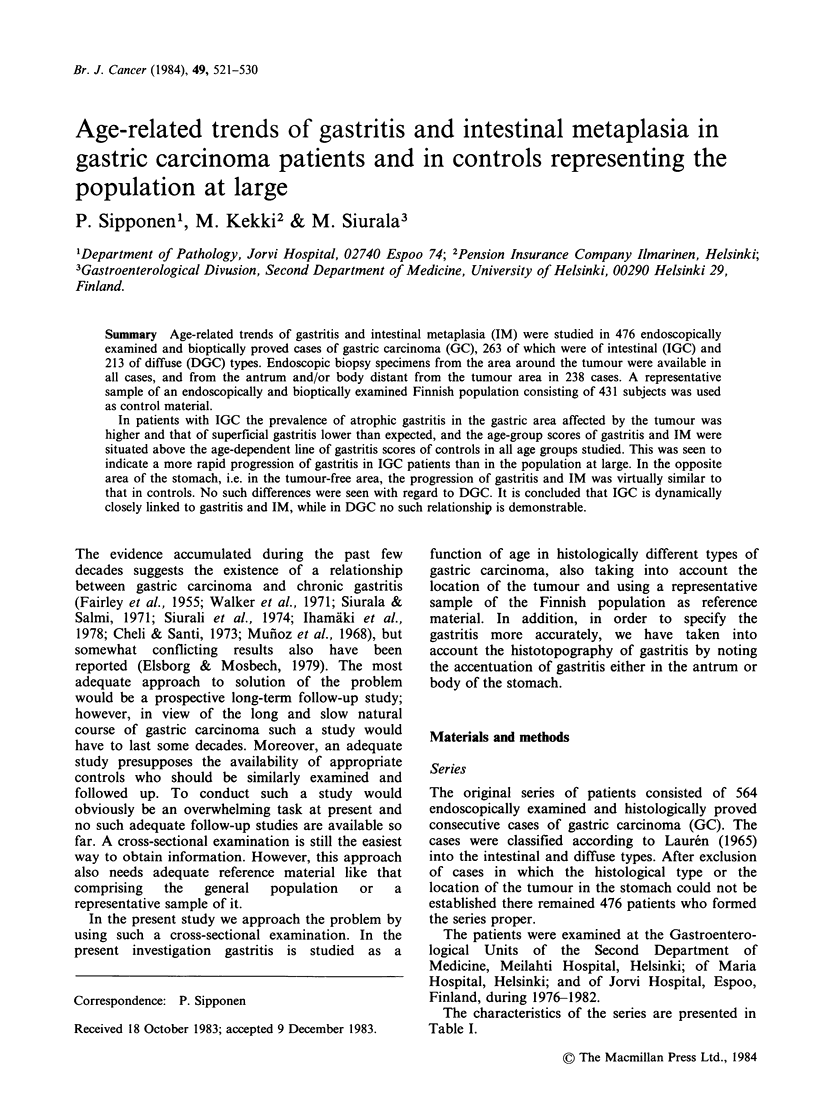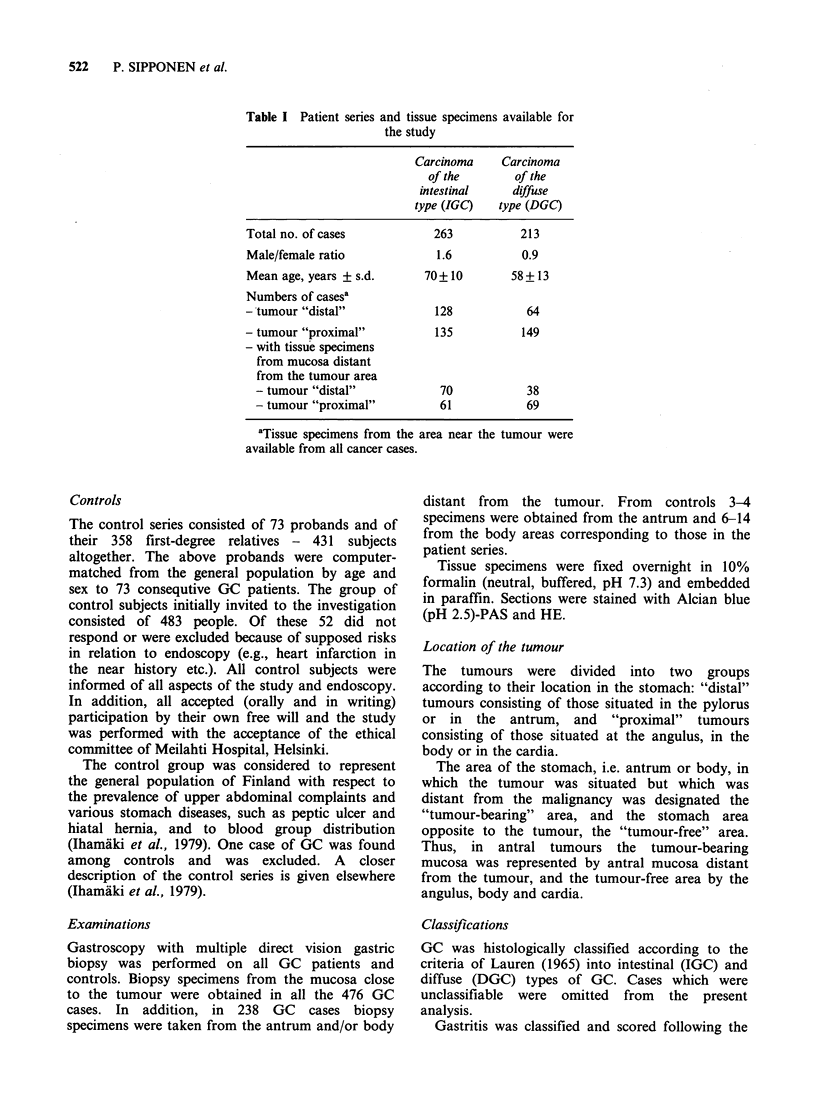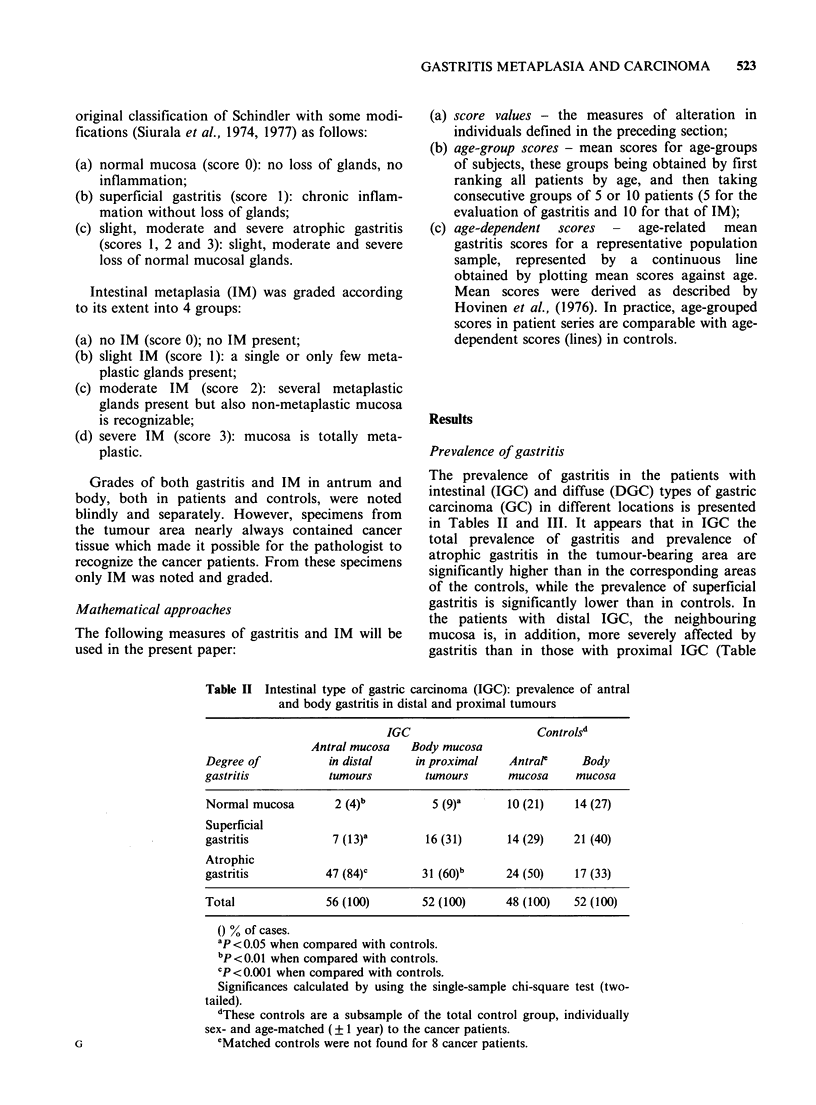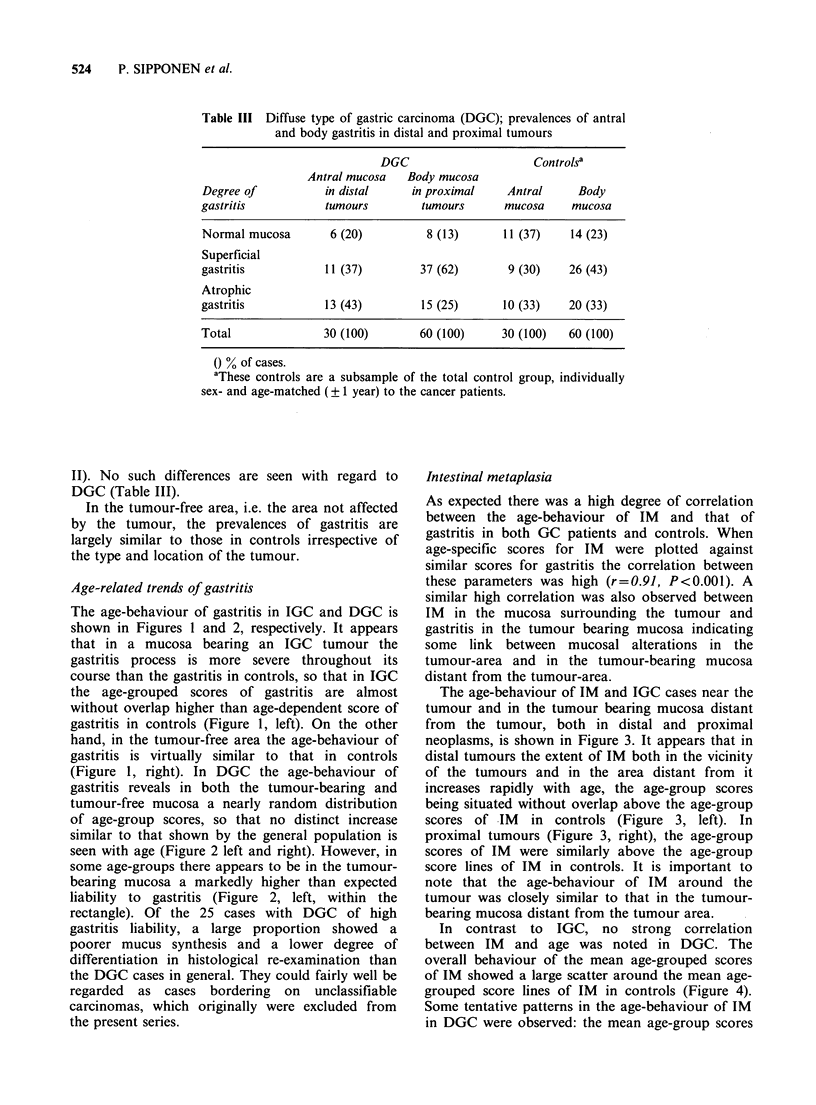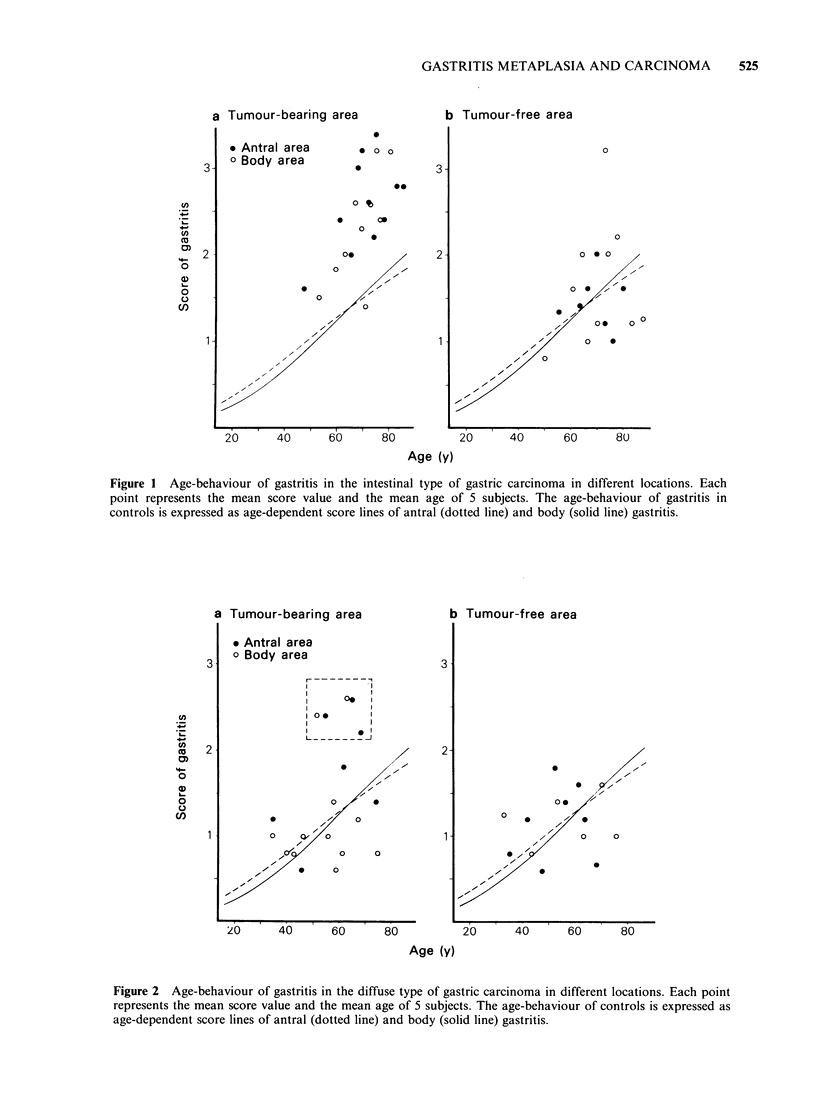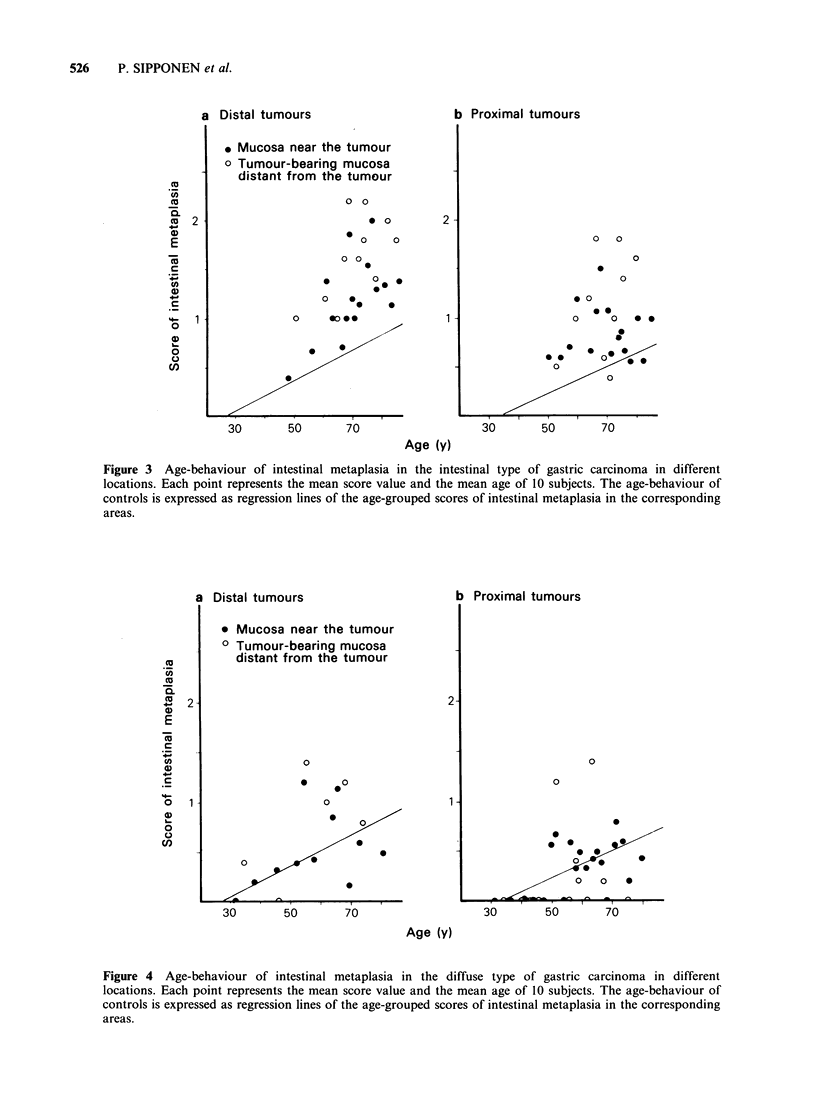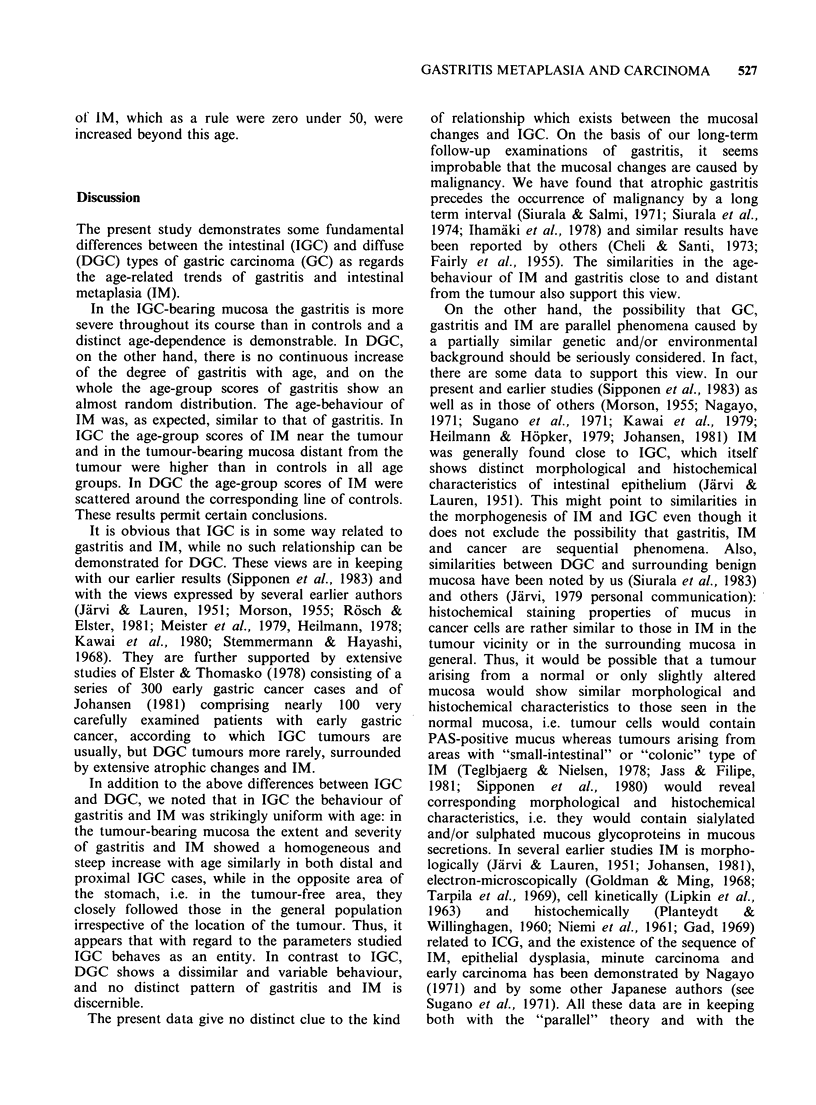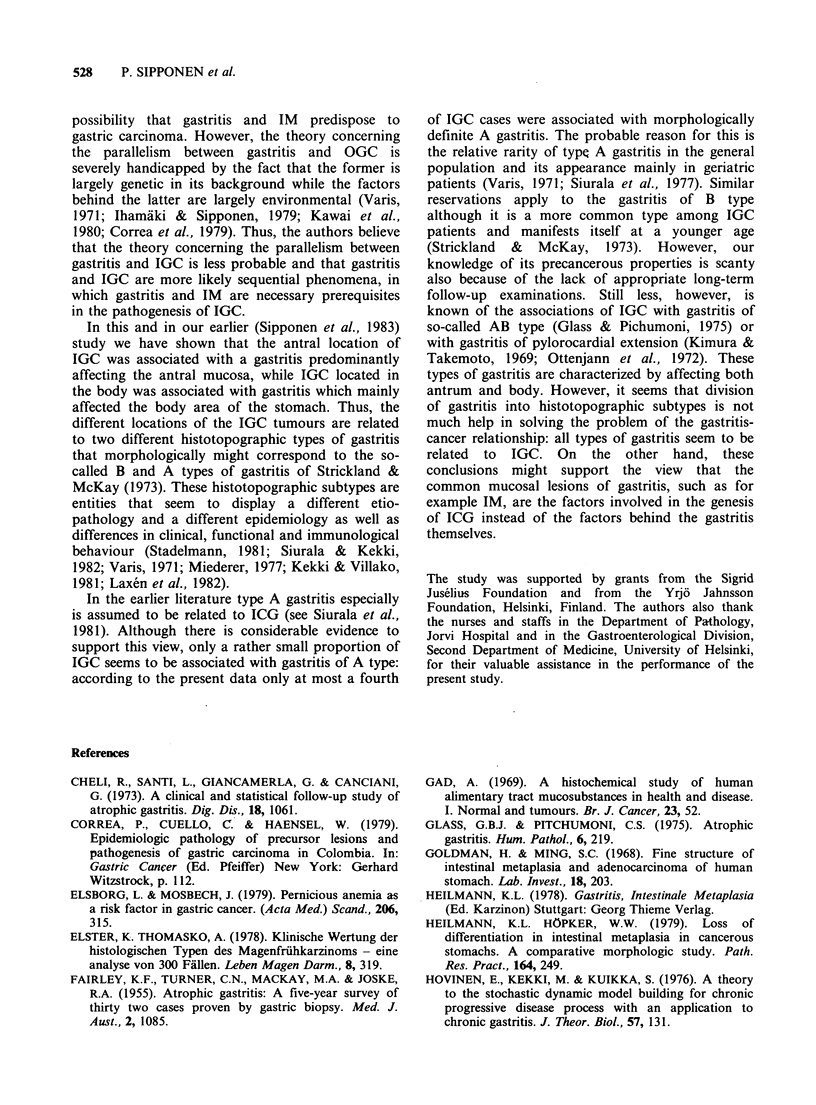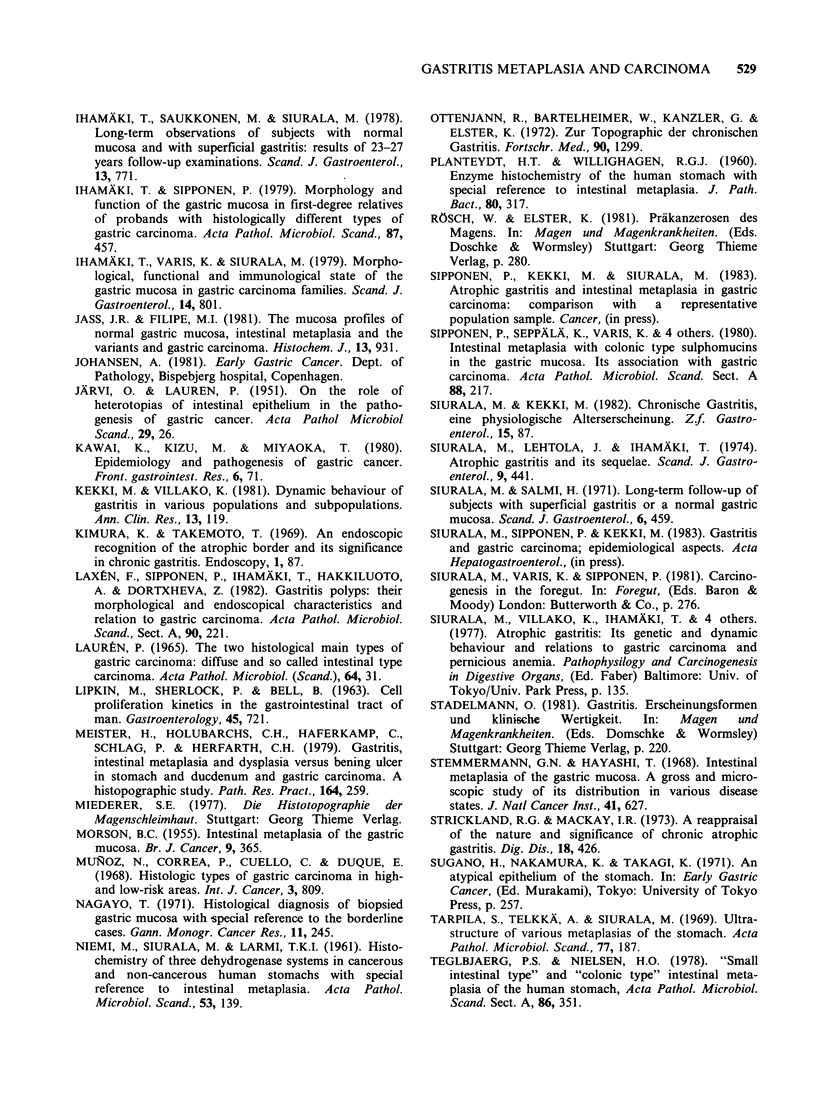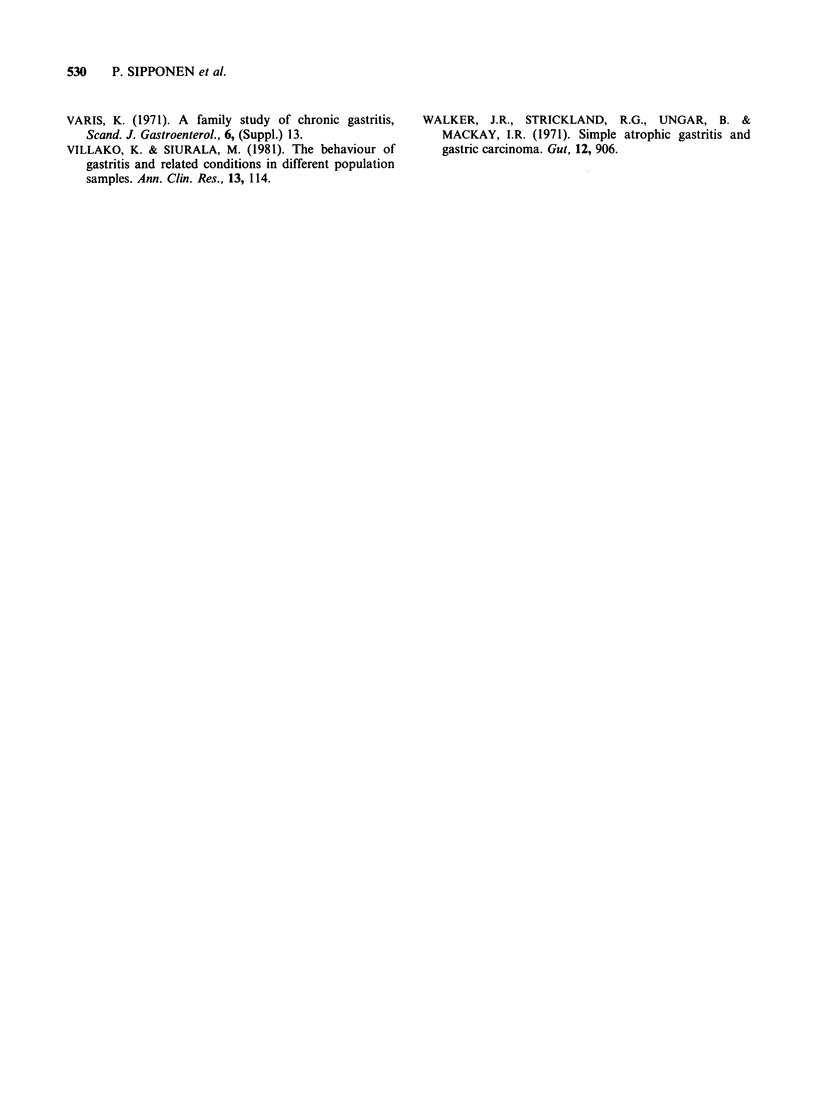# Age-related trends of gastritis and intestinal metaplasia in gastric carcinoma patients and in controls representing the population at large.

**DOI:** 10.1038/bjc.1984.80

**Published:** 1984-04

**Authors:** P. Sipponen, M. Kekki, M. Siurala

## Abstract

Age-related trends of gastritis and intestinal metaplasia (IM) were studied in 476 endoscopically examined and bioptically proved cases of gastric carcinoma (GC), 263 of which were of intestinal (IGC) and 213 of diffuse (DGC) types. Endoscopic biopsy specimens from the area around the tumour were available in all cases, and from the antrum and/or body distant from the tumour area in 238 cases. A representative sample of an endoscopically and bioptically examined Finnish population consisting of 431 subjects was used as control material. In patients with IGC the prevalence of atrophic gastritis in the gastric area affected by the tumour was higher and that of superficial gastritis lower than expected, and the age-group scores of gastritis and IM were situated above the age-dependent line of gastritis scores of controls in all age groups studied. This was seen to indicate a more rapid progression of gastritis in IGC patients than in the population at large. In the opposite area of the stomach, i.e. in the tumour-free area, the progression of gastritis and IM was virtually similar to that in controls. No such differences were seen with regard to DGC. It is concluded that IGC is dynamically closely linked to gastritis and IM, while in DGC no such relationship is demonstrable.


*Br. J. Cancer* (1984), **49**, 521–530

### Age-related trends of gastritis and intestinal metaplasia in gastric carcinoma patients and in controls representing the population at large

P. Sipponen[1], M. Kekki[2] & M. Siurala[3]

[1]*Department of Pathology, Jorvi Hospital, 02740 Espoo 74*; [2]*Pension Insurance Company Ilmarinen, Helsinki*; [3]*Gastroenterological Divusion, Second Department of Medicine, University of Helsinki, 00290 Helsinki 29, Finland.*

**Summary** Age-related trends of gastritis and intestinal metaplasia (IM) were studied in 476 endoscopically examined and bioptically proved cases of gastric carcinoma (GC), 263 of which were of intestinal (IGC) and 213 of diffuse (DGC) types. Endoscopic biopsy specimens from the area around the tumour were available in all cases, and from the antrum and/or body distant from the tumour area in 238 cases. A representative sample of an endoscopically and bioptically examined Finnish population consisting of 431 subjects was used as control material.

In patients with IGC the prevalence of atrophic gastritis in the gastric area affected by the tumour was higher and that of superficial gastritis lower than expected, and the age-group scores of gastritis and IM were situated above the age-dependent line of gastritis scores of controls in all age groups studied. This was seen to indicate a more rapid progression of gastritis in IGC patients than in the population at large. In the opposite area of the stomach, i.e. in the tumour-free area, the progression of gastritis and IM was virtually similar to that in controls. No such differences were seen with regard to DGC. It is concluded that IGC is dynamically closely linked to gastritis and IM, while in DGC no such relationship is demonstrable.

The evidence accumulated during the past few decades suggests the existence of a relationship between gastric carcinoma and chronic gastritis (Fairley *et al.*, 1955; Walker *et al.*, 1971; Siurala & Salmi, 1971; Siurali *et al.*, 1974; Ihamäki *et al.*, 1978; Cheli & Santi, 1973; Muñoz *et al.*, 1968), but somewhat conflicting results also have been reported (Elsborg & Mosbech, 1979). The most adequate approach to solution of the problem would be a prospective long-term follow-up study; however, in view of the long and slow natural course of gastric carcinoma such a study would have to last some decades. Moreover, an adequate study presupposes the availability of appropriate controls who should be similarly examined and followed up. To conduct such a study would obviously be an overwhelming task at present and no such adequate follow-up studies are available so far. A cross-sectional examination is still the easiest way to obtain information. However, this approach also needs adequate reference material like that comprising the general population or a representative sample of it.

In the present study we approach the problem by using such a cross-sectional examination. In the present investigation gastritis is studied as a function of age in histologically different types of gastric carcinoma, also taking into account the location of the tumour and using a representative sample of the Finnish population as reference material. In addition, in order to specify the gastritis more accurately, we have taken into account the histotopography of gastritis by noting the accentuation of gastritis either in the antrum or body of the stomach.

Correspondence: P. Sipponen

Received 18 October 1983; accepted 9 December 1983.

### Materials and methods

### *Series*

The original series of patients consisted of 564 endoscopically examined and histologically proved consecutive cases of gastric carcinoma (GC). The cases were classified according to Laurén (1965) into the intestinal and diffuse types. After exclusion of cases in which the histological type or the location of the tumour in the stomach could not be established there remained 476 patients who formed the series proper.

The patients were examined at the Gastroenterological Units of the Second Department of Medicine, Meilahti Hospital, Helsinki; of Maria Hospital, Helsinki; and of Jorvi Hospital, Espoo, Finland, during 1976–1982.

The characteristics of the series are presented in Table I.

© The Macmillan Press Ltd., 1984

**Table I** Patient series and tissue specimens available for the study

| | *Carcinoma of the intestinal type (IGC)* | *Carcinoma of the diffuse type (DGC)* |
|---|---|---|
| Total no. of cases | 263 | 213 |
| Male/female ratio | 1.6 | 0.9 |
| Mean age, years ± s.d. | 70±10 | 58±13 |
| Numbers of cases[a] | | |
| – tumour "distal" | 128 | 64 |
| – tumour "proximal" | 135 | 149 |
| – with tissue specimens from mucosa distant from the tumour area | | |
| – tumour "distal" | 70 | 38 |
| – tumour "proximal" | 61 | 69 |

[a]Tissue specimens from the area near the tumour were available from all cancer cases.

*Controls*

The control series consisted of 73 probands and of their 358 first-degree relatives – 431 subjects altogether. The above probands were computer-matched from the general population by age and sex to 73 consequtive GC patients. The group of control subjects initially invited to the investigation consisted of 483 people. Of these 52 did not respond or were excluded because of supposed risks in relation to endoscopy (e.g., heart infarction in the near history etc.). All control subjects were informed of all aspects of the study and endoscopy. In addition, all accepted (orally and in writing) participation by their own free will and the study was performed with the acceptance of the ethical committee of Meilahti Hospital, Helsinki.

The control group was considered to represent the general population of Finland with respect to the prevalence of upper abdominal complaints and various stomach diseases, such as peptic ulcer and hiatal hernia, and to blood group distribution (Ihamäki *et al.*, 1979). One case of GC was found among controls and was excluded. A closer description of the control series is given elsewhere (Ihamäki *et al.*, 1979).

*Examinations*

Gastroscopy with multiple direct vision gastric biopsy was performed on all GC patients and controls. Biopsy specimens from the mucosa close to the tumour were obtained in all the 476 GC cases. In addition, in 238 GC cases biopsy specimens were taken from the antrum and/or body distant from the tumour. From controls 3–4 specimens were obtained from the antrum and 6–14 from the body areas corresponding to those in the patient series.

Tissue specimens were fixed overnight in 10% formalin (neutral, buffered, pH 7.3) and embedded in paraffin. Sections were stained with Alcian blue (pH 2.5)-PAS and HE.

*Location of the tumour*

The tumours were divided into two groups according to their location in the stomach: "distal" tumours consisting of those situated in the pylorus or in the antrum, and "proximal" tumours consisting of those situated at the angulus, in the body or in the cardia.

The area of the stomach, i.e. antrum or body, in which the tumour was situated but which was distant from the malignancy was designated the "tumour-bearing" area, and the stomach area opposite to the tumour, the "tumour-free" area. Thus, in antral tumours the tumour-bearing mucosa was represented by antral mucosa distant from the tumour, and the tumour-free area by the angulus, body and cardia.

*Classifications*

GC was histologically classified according to the criteria of Lauren (1965) into intestinal (IGC) and diffuse (DGC) types of GC. Cases which were unclassifiable were omitted from the present analysis.

Gastritis was classified and scored following the original classification of Schindler with some modifications (Siurala *et al.*, 1974, 1977) as follows:

(a) normal mucosa (score 0): no loss of glands, no inflammation;
(b) superficial gastritis (score 1): chronic inflammation without loss of glands;
(c) slight, moderate and severe atrophic gastritis (scores 1, 2 and 3): slight, moderate and severe loss of normal mucosal glands.

Intestinal metaplasia (IM) was graded according to its extent into 4 groups:

(a) no IM (score 0); no IM present;
(b) slight IM (score 1): a single or only few metaplastic glands present;
(c) moderate IM (score 2): several metaplastic glands present but also non-metaplastic mucosa is recognizable;
(d) severe IM (score 3): mucosa is totally metaplastic.

Grades of both gastritis and IM in antrum and body, both in patients and controls, were noted blindly and separately. However, specimens from the tumour area nearly always contained cancer tissue which made it possible for the pathologist to recognize the cancer patients. From these specimens only IM was noted and graded.

*Mathematical approaches*

The following measures of gastritis and IM will be used in the present paper:

(a) *score values* – the measures of alteration in individuals defined in the preceding section;
(b) *age-group scores* – mean scores for age-groups of subjects, these groups being obtained by first ranking all patients by age, and then taking consecutive groups of 5 or 10 patients (5 for the evaluation of gastritis and 10 for that of IM);
(c) *age-dependent scores* – age-related mean gastritis scores for a representative population sample, represented by a continuous line obtained by plotting mean scores against age. Mean scores were derived as described by Hovinen *et al.*, (1976). In practice, age-grouped scores in patient series are comparable with age-dependent scores (lines) in controls.

### Results

*Prevalence of gastritis*

The prevalence of gastritis in the patients with intestinal (IGC) and diffuse (DGC) types of gastric carcinoma (GC) in different locations is presented in Tables II and III. It appears that in IGC the total prevalence of gastritis and prevalence of atrophic gastritis in the tumour-bearing area are significantly higher than in the corresponding areas of the controls, while the prevalence of superficial gastritis is significantly lower than in controls. In the patients with distal IGC, the neighbouring mucosa is, in addition, more severely affected by gastritis than in those with proximal IGC (Table

**Table II** Intestinal type of gastric carcinoma (IGC): prevalence of antral and body gastritis in distal and proximal tumours

| *Degree of gastritis* | *IGC* *Antral mucosa in distal tumours* | *Body mucosa in proximal tumours* | *Controls*[d] *Antral*[e] *mucosa* | *Body mucosa* |
|---|---|---|---|---|
| Normal mucosa | 2 (4)[b] | 5 (9)[a] | 10 (21) | 14 (27) |
| Superficial gastritis | 7 (13)[a] | 16 (31) | 14 (29) | 21 (40) |
| Atrophic gastritis | 47 (84)[c] | 31 (60)[b] | 24 (50) | 17 (33) |
| Total | 56 (100) | 52 (100) | 48 (100) | 52 (100) |

() % of cases.
[a]$P<0.05$ when compared with controls.
[b]$P<0.01$ when compared with controls.
[c]$P<0.001$ when compared with controls.
Significances calculated by using the single-sample chi-square test (two-tailed).
[d]These controls are a subsample of the total control group, individually sex- and age-matched ($\pm 1$ year) to the cancer patients.
[e]Matched controls were not found for 8 cancer patients.

**Table III** Diffuse type of gastric carcinoma (DGC); prevalences of antral and body gastritis in distal and proximal tumours

| *Degree of gastritis* | *DGC* *Antral mucosa in distal tumours* | *Body mucosa in proximal tumours* | *Controls*[a] *Antral mucosa* | *Body mucosa* |
|---|---|---|---|---|
| Normal mucosa | 6 (20) | 8 (13) | 11 (37) | 14 (23) |
| Superficial gastritis | 11 (37) | 37 (62) | 9 (30) | 26 (43) |
| Atrophic gastritis | 13 (43) | 15 (25) | 10 (33) | 20 (33) |
| Total | 30 (100) | 60 (100) | 30 (100) | 60 (100) |

() % of cases.
[a]These controls are a subsample of the total control group, individually sex- and age-matched (±1 year) to the cancer patients.

II). No such differences are seen with regard to DGC (Table III).

In the tumour-free area, i.e. the area not affected by the tumour, the prevalences of gastritis are largely similar to those in controls irrespective of the type and location of the tumour.

*Age-related trends of gastritis*

The age-behaviour of gastritis in IGC and DGC is shown in Figures 1 and 2, respectively. It appears that in a mucosa bearing an IGC tumour the gastritis process is more severe throughout its course than the gastritis in controls, so that in IGC the age-grouped scores of gastritis are almost without overlap higher than age-dependent score of gastritis in controls (Figure 1, left). On the other hand, in the tumour-free area the age-behaviour of gastritis is virtually similar to that in controls (Figure 1, right). In DGC the age-behaviour of gastritis reveals in both the tumour-bearing and tumour-free mucosa a nearly random distribution of age-group scores, so that no distinct increase similar to that shown by the general population is seen with age (Figure 2 left and right). However, in some age-groups there appears to be in the tumour-bearing mucosa a markedly higher than expected liability to gastritis (Figure 2, left, within the rectangle). Of the 25 cases with DGC of high gastritis liability, a large proportion showed a poorer mucus synthesis and a lower degree of differentiation in histological re-examination than the DGC cases in general. They could fairly well be regarded as cases bordering on unclassifiable carcinomas, which originally were excluded from the present series.

*Intestinal metaplasia*

As expected there was a high degree of correlation between the age-behaviour of IM and that of gastritis in both GC patients and controls. When age-specific scores for IM were plotted against similar scores for gastritis the correlation between these parameters was high ($r=0.91$, $P<0.001$). A similar high correlation was also observed between IM in the mucosa surrounding the tumour and gastritis in the tumour bearing mucosa indicating some link between mucosal alterations in the tumour-area and in the tumour-bearing mucosa distant from the tumour-area.

The age-behaviour of IM and IGC cases near the tumour and in the tumour bearing mucosa distant from the tumour, both in distal and proximal neoplasms, is shown in Figure 3. It appears that in distal tumours the extent of IM both in the vicinity of the tumours and in the area distant from it increases rapidly with age, the age-group scores being situated without overlap above the age-group scores of IM in controls (Figure 3, left). In proximal tumours (Figure 3, right), the age-group scores of IM were similarly above the age-group score lines of IM in controls. It is important to note that the age-behaviour of IM around the tumour was closely similar to that in the tumour-bearing mucosa distant from the tumour area.

In contrast to IGC, no strong correlation between IM and age was noted in DGC. The overall behaviour of the mean age-grouped scores of IM showed a large scatter around the mean age-grouped score lines of IM in controls (Figure 4). Some tentative patterns in the age-behaviour of IM in DGC were observed: the mean age-group scores

**Figure 1** Age-behaviour of gastritis in the intestinal type of gastric carcinoma in different locations. Each point represents the mean score value and the mean age of 5 subjects. The age-behaviour of gastritis in controls is expressed as age-dependent score lines of antral (dotted line) and body (solid line) gastritis.

**Figure 2** Age-behaviour of gastritis in the diffuse type of gastric carcinoma in different locations. Each point represents the mean score value and the mean age of 5 subjects. The age-behaviour of controls is expressed as age-dependent score lines of antral (dotted line) and body (solid line) gastritis.

**Figure 3** Age-behaviour of intestinal metaplasia in the intestinal type of gastric carcinoma in different locations. Each point represents the mean score value and the mean age of 10 subjects. The age-behaviour of controls is expressed as regression lines of the age-grouped scores of intestinal metaplasia in the corresponding areas.

**Figure 4** Age-behaviour of intestinal metaplasia in the diffuse type of gastric carcinoma in different locations. Each point represents the mean score value and the mean age of 10 subjects. The age-behaviour of controls is expressed as regression lines of the age-grouped scores of intestinal metaplasia in the corresponding areas.

of IM, which as a rule were zero under 50, were increased beyond this age.

### Discussion

The present study demonstrates some fundamental differences between the intestinal (IGC) and diffuse (DGC) types of gastric carcinoma (GC) as regards the age-related trends of gastritis and intestinal metaplasia (IM).

In the IGC-bearing mucosa the gastritis is more severe throughout its course than in controls and a distinct age-dependence is demonstrable. In DGC, on the other hand, there is no continuous increase of the degree of gastritis with age, and on the whole the age-group scores of gastritis show an almost random distribution. The age-behaviour of IM was, as expected, similar to that of gastritis. In IGC the age-group scores of IM near the tumour and in the tumour-bearing mucosa distant from the tumour were higher than in controls in all age groups. In DGC the age-group scores of IM were scattered around the corresponding line of controls. These results permit certain conclusions.

It is obvious that IGC is in some way related to gastritis and IM, while no such relationship can be demonstrated for DGC. These views are in keeping with our earlier results (Sipponen *et al.*, 1983) and with the views expressed by several earlier authors (Järvi & Lauren, 1951; Morson, 1955; Rösch & Elster, 1981; Meister *et al.*, 1979, Heilmann, 1978; Kawai *et al.*, 1980; Stemmermann & Hayashi, 1968). They are further supported by extensive studies of Elster & Thomasko (1978) consisting of a series of 300 early gastric cancer cases and of Johansen (1981) comprising nearly 100 very carefully examined patients with early gastric cancer, according to which IGC tumours are usually, but DGC tumours more rarely, surrounded by extensive atrophic changes and IM.

In addition to the above differences between IGC and DGC, we noted that in IGC the behaviour of gastritis and IM was strikingly uniform with age: in the tumour-bearing mucosa the extent and severity of gastritis and IM showed a homogeneous and steep increase with age similarly in both distal and proximal IGC cases, while in the opposite area of the stomach, i.e. in the tumour-free area, they closely followed those in the general population irrespective of the location of the tumour. Thus, it appears that with regard to the parameters studied IGC behaves as an entity. In contrast to IGC, DGC shows a dissimilar and variable behaviour, and no distinct pattern of gastritis and IM is discernible.

The present data give no distinct clue to the kind of relationship which exists between the mucosal changes and IGC. On the basis of our long-term follow-up examinations of gastritis, it seems improbable that the mucosal changes are caused by malignancy. We have found that atrophic gastritis precedes the occurrence of malignancy by a long term interval (Siurala & Salmi, 1971; Siurala *et al.*, 1974; Ihamäki *et al.*, 1978) and similar results have been reported by others (Cheli & Santi, 1973; Fairly *et al.*, 1955). The similarities in the age-behaviour of IM and gastritis close to and distant from the tumour also support this view.

On the other hand, the possibility that GC, gastritis and IM are parallel phenomena caused by a partially similar genetic and/or environmental background should be seriously considered. In fact, there are some data to support this view. In our present and earlier studies (Sipponen *et al.*, 1983) as well as in those of others (Morson, 1955; Nagayo, 1971; Sugano *et al.*, 1971; Kawai *et al.*, 1979; Heilmann & Höpker, 1979; Johansen, 1981) IM was generally found close to IGC, which itself shows distinct morphological and histochemical characteristics of intestinal epithelium (Järvi & Lauren, 1951). This might point to similarities in the morphogenesis of IM and IGC even though it does not exclude the possibility that gastritis, IM and cancer are sequential phenomena. Also, similarities between DGC and surrounding benign mucosa have been noted by us (Siurala *et al.*, 1983) and others (Järvi, 1979 personal communication): histochemical staining properties of mucus in cancer cells are rather similar to those in IM in the tumour vicinity or in the surrounding mucosa in general. Thus, it would be possible that a tumour arising from a normal or only slightly altered mucosa would show similar morphological and histochemical characteristics to those seen in the normal mucosa, i.e. tumour cells would contain PAS-positive mucus whereas tumours arising from areas with "small-intestinal" or "colonic" type of IM (Teglbjaerg & Nielsen, 1978; Jass & Filipe, 1981; Sipponen *et al.*, 1980) would reveal corresponding morphological and histochemical characteristics, i.e. they would contain sialylated and/or sulphated mucous glycoproteins in mucous secretions. In several earlier studies IM is morphologically (Järvi & Lauren, 1951; Johansen, 1981), electron-microscopically (Goldman & Ming, 1968; Tarpila *et al.*, 1969), cell kinetically (Lipkin *et al.*, 1963) and histochemically (Planteydt & Willinghagen, 1960; Niemi *et al.*, 1961; Gad, 1969) related to ICG, and the existence of the sequence of IM, epithelial dysplasia, minute carcinoma and early carcinoma has been demonstrated by Nagayo (1971) and by some other Japanese authors (see Sugano *et al.*, 1971). All these data are in keeping both with the "parallel" theory and with the possibility that gastritis and IM predispose to gastric carcinoma. However, the theory concerning the parallelism between gastritis and OGC is severely handicapped by the fact that the former is largely genetic in its background while the factors behind the latter are largely environmental (Varis, 1971; Ihamäki & Sipponen, 1979; Kawai *et al.*, 1980; Correa *et al.*, 1979). Thus, the authors believe that the theory concerning the parallelism between gastritis and IGC is less probable and that gastritis and IGC are more likely sequential phenomena, in which gastritis and IM are necessary prerequisites in the pathogenesis of IGC.

In this and in our earlier (Sipponen *et al.*, 1983) study we have shown that the antral location of IGC was associated with a gastritis predominantly affecting the antral mucosa, while IGC located in the body was associated with gastritis which mainly affected the body area of the stomach. Thus, the different locations of the IGC tumours are related to two different histotopographic types of gastritis that morphologically might correspond to the so-called B and A types of gastritis of Strickland & McKay (1973). These histotopographic subtypes are entities that seem to display a different etio-pathology and a different epidemiology as well as differences in clinical, functional and immunological behaviour (Stadelmann, 1981; Siurala & Kekki, 1982; Varis, 1971; Miederer, 1977; Kekki & Villako, 1981; Laxén *et al.*, 1982).

In the earlier literature type A gastritis especially is assumed to be related to ICG (see Siurala *et al.*, 1981). Although there is considerable evidence to support this view, only a rather small proportion of IGC seems to be associated with gastritis of A type: according to the present data only at most a fourth of IGC cases were associated with morphologically definite A gastritis. The probable reason for this is the relative rarity of type A gastritis in the general population and its appearance mainly in geriatric patients (Varis, 1971; Siurala *et al.*, 1977). Similar reservations apply to the gastritis of B type although it is a more common type among IGC patients and manifests itself at a younger age (Strickland & McKay, 1973). However, our knowledge of its precancerous properties is scanty also because of the lack of appropriate long-term follow-up examinations. Still less, however, is known of the associations of IGC with gastritis of so-called AB type (Glass & Pichumoni, 1975) or with gastritis of pylorocardial extension (Kimura & Takemoto, 1969; Ottenjann *et al.*, 1972). These types of gastritis are characterized by affecting both antrum and body. However, it seems that division of gastritis into histotopographic subtypes is not much help in solving the problem of the gastritis-cancer relationship: all types of gastritis seem to be related to IGC. On the other hand, these conclusions might support the view that the common mucosal lesions of gastritis, such as for example IM, are the factors involved in the genesis of ICG instead of the factors behind the gastritis themselves.

The study was supported by grants from the Sigrid Jusélius Foundation and from the Yrjö Jahnsson Foundation, Helsinki, Finland. The authors also thank the nurses and staffs in the Department of Pathology, Jorvi Hospital and in the Gastroenterological Division, Second Department of Medicine, University of Helsinki, for their valuable assistance in the performance of the present study.

### References

CHELI, R., SANTI, L., GIANCAMERLA, G. & CANCIANI, G. (1973). A clinical and statistical follow-up study of atrophic gastritis. *Dig. Dis.*, **18**, 1061.

CORREA, P., CUELLO, C. & HAENSEL, W. (1979). Epidemiologic pathology of precursor lesions and pathogenesis of gastric carcinoma in Colombia. In: *Gastric Cancer* (Ed. Pfeiffer) New York: Gerhard Witzstrock, p. 112.

ELSBORG, L. & MOSBECH, J. (1979). Pernicious anemia as a risk factor in gastric cancer. (*Acta Med.*) *Scand.*, **206**, 315.

ELSTER, K. THOMASKO, A. (1978). Klinische Wertung der histologischen Typen des Magenfrühkarzinoms – eine analyse von 300 Fällen. *Leben Magen Darm.*, **8**, 319.

FAIRLEY, K.F., TURNER, C.N., MACKAY, M.A. & JOSKE, R.A. (1955). Atrophic gastritis: A five-year survey of thirty two cases proven by gastric biopsy. *Med. J. Aust.*, **2**, 1085.

GAD, A. (1969). A histochemical study of human alimentary tract mucosubstances in health and disease. I. Normal and tumours. *Br. J. Cancer*, **23**, 52.

GLASS, G.B.J. & PITCHUMONI, C.S. (1975). Atrophic gastritis. *Hum. Pathol.*, **6**, 219.

GOLDMAN, H. & MING, S.C. (1968). Fine structure of intestinal metaplasia and adenocarcinoma of human stomach. *Lab. Invest.*, **18**, 203.

HEILMANN, K.L. (1978). *Gastritis, Intestinale Metaplasia* (Ed. Karzinon) Stuttgart: Georg Thieme Verlag.

HEILMANN, K.L. HÖPKER, W.W. (1979). Loss of differentiation in intestinal metaplasia in cancerous stomachs. A comparative morphologic study. *Path. Res. Pract.*, **164**, 249.

HOVINEN, E., KEKKI, M. & KUIKKA, S. (1976). A theory to the stochastic dynamic model building for chronic progressive disease process with an application to chronic gastritis. *J. Theor. Biol.*, **57**, 131.

IHAMÄKI, T., SAUKKONEN, M. & SIURALA, M. (1978). Long-term observations of subjects with normal mucosa and with superficial gastritis: results of 23–27 years follow-up examinations. *Scand. J. Gastroenterol.*, **13**, 771.

IHAMÄKI, T. & SIPPONEN, P. (1979). Morphology and function of the gastric mucosa in first-degree relatives of probands with histologically different types of gastric carcinoma. *Acta Pathol. Microbiol. Scand.*, **87**, 457.

IHAMÄKI, T., VARIS, K. & SIURALA, M. (1979). Morphological, functional and immunological state of the gastric mucosa in gastric carcinoma families. *Scand. J. Gastroenterol.*, **14**, 801.

JASS, J.R. & FILIPE, M.I. (1981). The mucosa profiles of normal gastric mucosa, intestinal metaplasia and the variants and gastric carcinoma. *Histochem. J.*, **13**, 931.

JOHANSEN, A. (1981). *Early Gastric Cancer*. Dept. of Pathology, Bispebjerg hospital, Copenhagen.

JÄRVI, O. & LAUREN, P. (1951). On the role of heterotopias of intestinal epithelium in the pathogenesis of gastric cancer. *Acta Pathol Microbiol Scand.*, **29**, 26.

KAWAI, K., KIZU, M. & MIYAOKA, T. (1980). Epidemiology and pathogenesis of gastric cancer. *Front. gastrointest. Res.*, **6**, 71.

KEKKI, M. & VILLAKO, K. (1981). Dynamic behaviour of gastritis in various populations and subpopulations. *Ann. Clin. Res.*, **13**, 119.

KIMURA, K. & TAKEMOTO, T. (1969). An endoscopic recognition of the atrophic border and its significance in chronic gastritis. Endoscopy, **1**, 87.

LAXÉN, F., SIPPONEN, P., IHAMÄKI, T., HAKKILUOTO, A. & DORTXHEVA, Z. (1982). Gastritis polyps: their morphological and endoscopical characteristics and relation to gastric carcinoma. *Acta Pathol. Microbiol. Scand.*, Sect. A, **90**, 221.

LAURÉN, P. (1965). The two histological main types of gastric carcinoma: diffuse and so called intestinal type carcinoma. *Acta Pathol. Microbiol. (Scand.)*, **64**, 31.

LIPKIN, M., SHERLOCK, P. & BELL, B. (1963). Cell proliferation kinetics in the gastrointestinal tract of man. *Gastroenterology*, **45**, 721.

MEISTER, H., HOLUBARCHS, C.H., HAFERKAMP, C., SCHLAG, P. & HERFARTH, C.H. (1979). Gastritis, intestinal metaplasia and dysplasia versus bening ulcer in stomach and ducdenum and gastric carcinoma. A histopographic study. *Path. Res. Pract.*, **164**, 259.

MIEDERER, S.E. (1977). *Die Histotopographie der Magenschleimhaut*. Stuttgart: Georg Thieme Verlag.

MORSON, B.C. (1955). Intestinal metaplasia of the gastric mucosa. *Br. J. Cancer*, **9**, 365.

MUÑOZ, N., CORREA, P., CUELLO, C. & DUQUE, E. (1968). Histologic types of gastric carcinoma in high- and low-risk areas. *Int. J. Cancer*, **3**, 809.

NAGAYO, T. (1971). Histological diagnosis of biopsied gastric mucosa with special reference to the borderline cases. *Gann. Monogr. Cancer Res.*, **11**, 245.

NIEMI, M., SIURALA, M. & LARMI, T.K.I. (1961). Histochemistry of three dehydrogenase systems in cancerous and non-cancerous human stomachs with special reference to intestinal metaplasia. *Acta Pathol. Microbiol. Scand.*, **53**, 139.

OTTENJANN, R., BARTELHEIMER, W., KANZLER, G. & ELSTER, K. (1972). Zur Topographic der chronischen Gastritis. *Fortschr. Med.*, **90**, 1299.

PLANTEYDT, H.T. & WILLIGHAGEN, R.G.J. (1960). Enzyme histochemistry of the human stomach with special reference to intestinal metaplasia. *J. Path. Bact.*, **80**, 317.

RÖSCH, W. & ELSTER, K. (1981). Präkanzerosen des Magens. In: *Magen und Magenkrankheiten*. (Eds. Doschke & Wormsley) Stuttgart: Georg Thieme Verlag, p. 280.

SIPPONEN, P., KEKKI, M. & SIURALA, M. (1983). Atrophic gastritis and intestinal metaplasia in gastric carcinoma: comparison with a representative population sample. *Cancer*, (in press).

SIPPONEN, P., SEPPÄLÄ, K., VARIS, K. & 4 others. (1980). Intestinal metaplasia with colonic type sulphomucins in the gastric mucosa. Its association with gastric carcinoma. *Acta Pathol. Microbiol. Scand.* Sect. A **88**, 217.

SIURALA, M. & KEKKI, M. (1982). Chronische Gastritis, eine physiologische Alterserscheinung. *Z.f. Gastroenterol.*, **15**, 87.

SIURALA, M., LEHTOLA, J. & IHAMÄKI, T. (1974). Atrophic gastritis and its sequelae. *Scand. J. Gastroenterol.*, **9**, 441.

SIURALA, M. & SALMI, H. (1971). Long-term follow-up of subjects with superficial gastritis or a normal gastric mucosa. *Scand. J. Gastroenterol.*, **6**, 459.

SIURALA, M., SIPPONEN, P. & KEKKI, M. (1983). Gastritis and gastric carcinoma; epidemiological aspects. *Acta Hepatogastroenterol.*, (in press).

SIURALA, M., VARIS, K. & SIPPONEN, P. (1981). Carcinogenesis in the foregut. In: *Foregut*, (Eds. Baron & Moody) London: Butterworth & Co., p. 276.

SIURALA, M., VILLAKO, K., IHAMÄKI, T. & 4 others. (1977). Atrophic gastritis: Its genetic and dynamic behaviour and relations to gastric carcinoma and pernicious anemia. *Pathophysilogy and Carcinogenesis in Digestive Organs*, (Ed. Faber) Baltimore: Univ. of Tokyo/Univ. Park Press, p. 135.

STADELMANN, O. (1981). Gastritis. Erscheinungsformen und klinische Wertigkeit. In: *Magen und Magenkrankheiten*. (Eds. Domschke & Wormsley) Stuttgart: Georg Thieme Verlag, p. 220.

STEMMERMANN, G.N. & HAYASHI, T. (1968). Intestinal metaplasia of the gastric mucosa. A gross and microscopic study of its distribution in various disease states. *J. Natl Cancer Inst.*, **41**, 627.

STRICKLAND, R.G. & MACKAY, I.R. (1973). A reappraisal of the nature and significance of chronic atrophic gastritis. *Dig. Dis.*, **18**, 426.

SUGANO, H., NAKAMURA, K. & TAKAGI, K. (1971). An atypical epithelium of the stomach. In: *Early Gastric Cancer*, (Ed. Murakami), Tokyo: University of Tokyo Press, p. 257.

TARPILA, S., TELKKÄ, A. & SIURALA, M. (1969). Ultrastructure of various metaplasias of the stomach. *Acta Pathol. Microbiol. Scand.*, **77**, 187.

TEGLBJAERG, P.S. & NIELSEN, H.O. (1978). "Small intestinal type" and "colonic type" intestinal metaplasia of the human stomach, *Acta Pathol. Microbiol. Scand.* Sect. A, **86**, 351.

VARIS, K. (1971). A family study of chronic gastritis, *Scand. J. Gastroenterol.*, **6,** (Suppl.) 13.

VILLAKO, K. & SIURALA, M. (1981). The behaviour of gastritis and related conditions in different population samples. *Ann. Clin. Res.*, **13,** 114.

WALKER, J.R., STRICKLAND, R.G., UNGAR, B. & MACKAY, I.R. (1971). Simple atrophic gastritis and gastric carcinoma. *Gut,* **12,** 906.